# HLA-DPB1 rs9277535 polymorphism is associated with rheumatoid arthritis risk in a Chinese Han population

**DOI:** 10.18632/aging.202864

**Published:** 2021-04-19

**Authors:** Zhicheng Yang, Weixi Liu, Ting Yan, Ruiping Liu

**Affiliations:** 1 Department of Orthopedics, The Affiliated Changzhou No.2 People’s Hospital of Nanjing Medical University, Changzhou 213003, China

**Keywords:** HLA-DPB1, polymorphism, rheumatoid arthritis

## Abstract

We used a custom-by-design 48-Plex single nucleotide polymorphism scan™ kit to investigate the relationship between susceptibility to rheumatoid arthritis (RA) and HLA-DPB1 rs9277535 polymorphism in 805 RA patients and 1095 healthy controls from the Chinese Han population. Blood plasma levels of HLA-DPB1 were also examined using enzyme-linked immunosorbent assays in 170 RA patients and 170 matched control individuals. Quantitative reverse transcription PCR (qRT-PCR) was used to evaluate relative HLA-DPB1 mRNA levels in these blood samples as well. The results indicated that some HLA-DPB1 rs9277535 polymorphisms decreased RA susceptibility. Stratified analysis indicated that risk of RA decreased specifically in women and those who were at least 55 years old. In addition, the AG and GG+AG genotypes were associated with CRP status, ACPA status, and ESR in RA patients when the AA genotype was used as the reference group. Furthermore, average HLA-DPB1 plasma levels were increased in RA patients, and HLA-DPB1 plasma levels and mRNA expression were lower in those with the GG genotype than in those with the AA genotype. These results indicate that HLA-DPB1 rs9277535 polymorphism is associated with a decreased risk of RA in the Chinese population.

## INTRODUCTION

Rheumatoid arthritis (RA) is an autoimmune, inflammation-related, chronic disorder primarily affecting the joints [1]. The average prevalence of RA globally is 0.5-1.0%, and genetic factors account for 60% of the risk of developing RA [1]. RA is 2 to 3 times more common in women than in men and can develop at any age [2]. Though the cause of RA remains unclear, genetics, female sex, and environmental factors are known risk factors in RA development [3]. Furthermore, as comprehensively demonstrated by recent studies, 48 novel loci including three HLA loci are associated with RA susceptibility [4].

The three DP alleles, which are found in the HLA region, form heterodimeric glycoproteins consisting of A- and B-chains [5]. These A- and B-chains are coded separately by the DPB1 and DPA1 alleles on the sixth chromosome, and the nucleotide sequence of the DPB1 exon varies most significantly, leading to allele polymorphism and diverse phenotypes [6]. Increasing evidence indicates that HLA-DR in general, and the HLA-DRB1 locus in particular, is a critical genetic risk factor for RA [7–9]. The roles of HLA-DP genes in psoriasis (POS), systemic lupus erythematosus (SLE), and dermatomyositis (DM) have also been investigated [10–12]. Furukawa et al. suggested that increased DPB1 allele frequency was associated with the development and progression of SLE [11]. In addition, Kamatani et al*.* reported that HLA-DP locus variants were related to chronic hepatitis B in Asians [13].

Rs9277535, which is located in the HLA-DPB1 3′-UTR, affects HLA-DP mRNA expression. Single nucleotide polymorphisms (SNPs) in the HLA-DPB1 gene are likely to impact its expression and function, potentially contributing to RA susceptibility. Given the importance of HLA-DPB1 gene polymorphism in RA, we performed this clinical study including 805 RA patients and 1095 healthy controls to examine whether HLA-DPB1 gene rs9277535 polymorphisms were associated with RA risk and clinical characteristics in a Chinese population.

## RESULTS

### Subject characteristics

Subject characteristics are summarized in Table 1. The RA patient and healthy control groups were well matched regarding age and sex (*P* = 0.169 and *P* = 0.070, respectively). The HLA-DPB1 rs9277535 genotype distribution for all study subjects is shown in Table 2. HWE analysis revealed no differences in the control group (*P* = 0.251).

**Table 1:** Patient demographics and risk factors in rheumatoid arthritis, all subjects.

Variable^*^	**Cases (n=805)**	**Controls (n=1095)**	***P***
Age (years)	55.92 (±15.10)	55.01 (±13.01)	0.169
Female/male	603/202	859/236	0.070
Age at onset, years, mean ± SD	45.97 (±12.31)	—	—
Disease duration, years, mean ± SD	10.04 (±9.66)	—	—
Treatment duration, years, mean ± SD	9.22 (±9.10)	—	—
RF-positive, no. (%)	643 (79.88%)	—	—
ACPA positive, no. (%)	451 (56.02%)	—	—
CRP-positive, no. (%)	470 (58.39%)	—	—
ESR, mm/h	35.90 (±28.87)	—	—
DAS28	4.49 (±1.55)	—	—
Functional class, no. (%)		—	—
I	79 (9.81%)	—	—
II	353 (44.85%)	—	—
III	314 (39.01%)	—	—
IV	59 (7.33%)	—	—
HLA-DPB1 levels^**^ (pg/ml)	161.52 ± 45.73	145.59 ± 48.04	**0.002**

^*^RF: Rheumatoid factor; ACPA: Anti-cyclic citrullinated peptide antibodies; CRP: C-reactive protein; ESR: Erythrocyte sedimentation rate; DAS28: RA disease activity score. ^**^ HLA-DPB1 levels come from samples collected recently (170 RA cases to 170 controls; age and gender match well: *P* of age is 0.075, *P* of gender is 0.390). Bold values are statistically significant (*P* < 0.05).

**Table 2:** Logistic regression analysis of associations between *HLA-DPB1* polymorphism and risk of rheumatoid arthritis.

**Genotype**	**Cases (n=805)**			**Controls (n=1095)**		**OR (95% CI)**	***P***
	**n**	**%**		**n**	**%**		
*HLA-DPB1* rs9277535							
AA	250	31.1		205	18.7	1.00	—
AG	332	41.2		559	51.1	**0.49(0.39-0.61)**	**0.000**
GG	223	27.7		331	30.2	**0.55(0.43-0.71)**	**0.000**
GG+AG	555	68.9		890	81.3	**0.51(0.41-0.63)**	**0.000**
AA+AG	582	72.3		764	69.8	1.00	—
GG	223	27.7		331	30.2	0.88(0.72-1.08)	0.231
G allele	778	48.3		1221	55.8	**0.74(0.65**-**0.84)**	**0.000**

The genotyping was successful in 805 cases and 1095 controls for *HLA-DPB1* rs9277535.

### Associations between HLA-DPB1 rs9277535 polymorphism and RA risk

The GG and AG genotypes were associated with a significantly reduced risk of RA (GG vs. AA: OR = 0.55, 95%CI = 0.43 - 0.71, *P* = 0.000; AG vs. AA: OR = 0.49, 95%CI = 0.39 - 0.61, *P* = 0.000). In addition, the GG+AG genotype was associated with a reduced RA risk compared to the AA genotype (GG+AG vs. AA: OR = 0.51, 95%CI = 0.41 - 0.63, *P* = 0.000) (Table 2). The G allele was also associated with a reduced risk of RA (G vs. A: OR = 0.74, 95%CI = 0.65-0.84, *P* = 0.000).

### Stratification analyses of HLA-DPB1 rs9277535 polymorphism and RA risk

Stratified analyses indicated that HLA-DPB1 rs9277535 polymorphism decreased RA risk in women and in those at least 55 years old. (Table 3). In addition, the AG and GG+AG genotypes were associated with CRP status, ACPA status, and ESR in RA patients when the AA genotype was used as the reference group (Table 4). No relationships were identified between HLA-DPB1 plasma concentrations and RF, ACPA, CRP, ESR, DAS28, or functional class (Table 5).

**Table 3:** Stratified analyses between *HLA-DPB1* rs9277535 polymorphism and the risk of rheumatoid arthritis.

**Variable**	**rs9277535 (case/control)**			**AG vs. AA**	**GG vs. AA**	**GG vs. AG+AA**	**AG+GG vs. AA**
	**AA**	**AG**	**GG**				
Sex							
Male	60/58	81/121	61/57	0.65(0.41-1.02); 0.062	1.04(0.62-1.72); 0.896	0.36(0.89-2.07); 0.155	0.77(0.51-1.18); 0.228
Female	190/147	251/438	162/274	**0.44(0.34-0.58); 0.000**	**0.46(0.34-0.61);0.000**	**0.78(0.62-0.99);0.038**	**0.45(0.35-0.58); 0.000**
Age (years)							
<55	90/104	172/271	93/148	0.73 (0.52-1.03); 0.074	0.73(0.50-1.07);0.101	0.90(0.66-1.22);0.494	0.96(0.69-1.33); 0.806
≥55	160/101	160/288	130/183	**0.35(0.26-0.48); 0.000**	**0.45(0.32-0.63); 0.000**	0.86 (0.66-1.13); 0.285	**0.39(0.29-0.52); 0.000**

Bold values are statistically significant (*P* <0.05).

**Table 4:** The associations between *HLA-DPB1* rs9277535 polymorphism and clinical characteristics of rheumatoid arthritis.

**Characteristics**	**Genotype distributions**	**AG**	**GG**	**AG+GG**
	**AA**			
CRP status				
Positive / Negative	165/85	174/158	131/92	305/250
OR (95%CI); *P*-value	1.0 (reference)	**0.57(0.40-0.80); 0.001**	0.73(0.51-1.07); 0.104	**0.63(0.46-0.86); 0.003**
ACPA status				
Positive / Negative	157/93	172/160	122/101	294/261
OR (95%CI); *P*-value	1.0 (reference)	**0.64(0.46-0.89);** **0.008**	0.72(0.50-1.03); 0.074	**0.67(0.49-0.91); 0.009**
RF status				
Positive / Negative	200/50	268/64	175/48	443/112
OR (95%CI); *P*-value	1.0 (reference)	1.05(0.69-1.58); 0.828	0.91(0.58-1.42); 0.683	0.99(0.68-1.44); 0.953
ESR (mm/h)				
≥25.00/<25.00	159/91	181/151	123/100	304/251
OR (95%CI); *P*-value	1.0 (reference)	**0.69(0.49-0.96); 0.028**	0.70(0.49-1.02); 0.062	**0.69(0.51-0.94); 0.019**
DAS28				
≥3.20/<3.20	197/53	255/77	173/50	428/127
OR (95%CI); *P*-value	1.0 (reference)	0.89(0.60-1.32); 0.568	0.93(0.60-1.44); 0.748	0.91(0.63-1.30); 0.596
Functional class				
III+IV / I+II	106/144	154/178	113/110	267/288
OR (95%CI); *P*-value	1.0 (reference)	1.18(0.84-1.64); 0.338	1.40(0.97-2.01); 0.072	1.26(0.93-1.70); 0.133

Bold values are statistically significant (*P* <0.05).

**Table 5:** Stratification of association between plasma levels of HLA-DPB1 and other biomarkers.

**Variable**	**Case**	**Mean ± SD**	***P***
RF status			
Negative	34	162.27 ± 42.11	0.915
Positive	136	161.33 ± 46.74	
ACPA			
Negative	57	157.94 ± 53.79	0.509
Positive	113	163.32 ± 41.22	
CRP status			
Negative	64	167.55 ± 43.52	0.183
Positive	106	157.88 ± 46.84	
ESR (mm/h)			
< 25.00	78	159.04 ± 44.55	0.517
≥ 25.00	92	163.62 ± 46.85	
DAS28			
< 3.20	43	167.77 ± 42.38	0.301
≥ 3.20	127	159.41 ± 46.78	
Functional class			
I + II	91	162.91 ± 43.48	0.673
III + IV	79	159.92 ± 48.42	

RF: rheumatoid factor; ACPA: anti-cyclic citrullinated peptide antibodies; CRP: C-reactive protein; ESR: erythrocyte sedimentation rate; DAS28: RA disease activity score; OR: odds ratios; CI: confidence intervals.

### Associations between HLA-DPB1 rs9277535 genotype, plasma levels, and mRNA expression in RA

**Figure 1:**
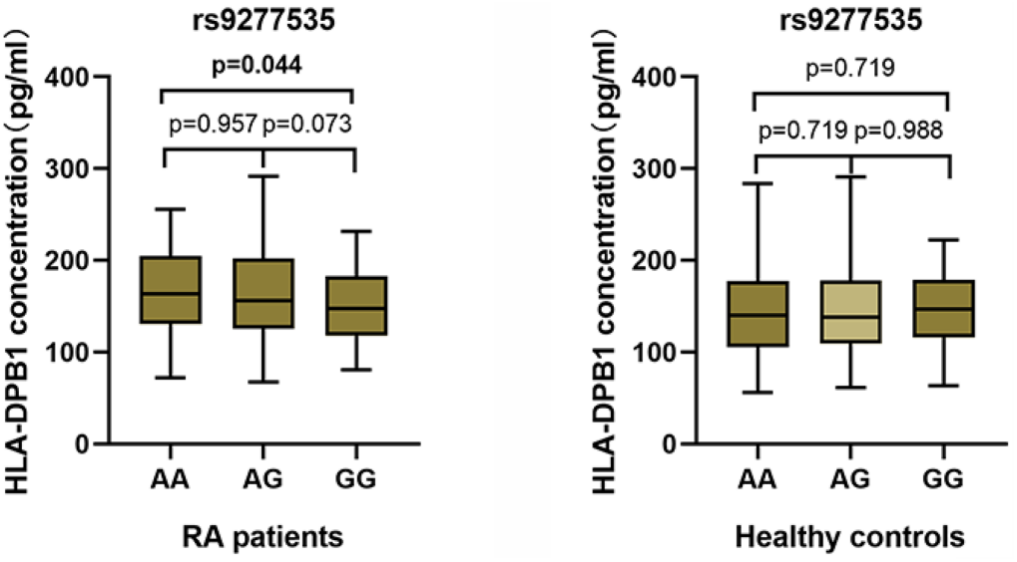
Associations between HLA-DPB1 plasma levels and genotype frequency in RA patients and healthy controls.

**Figure 2:**
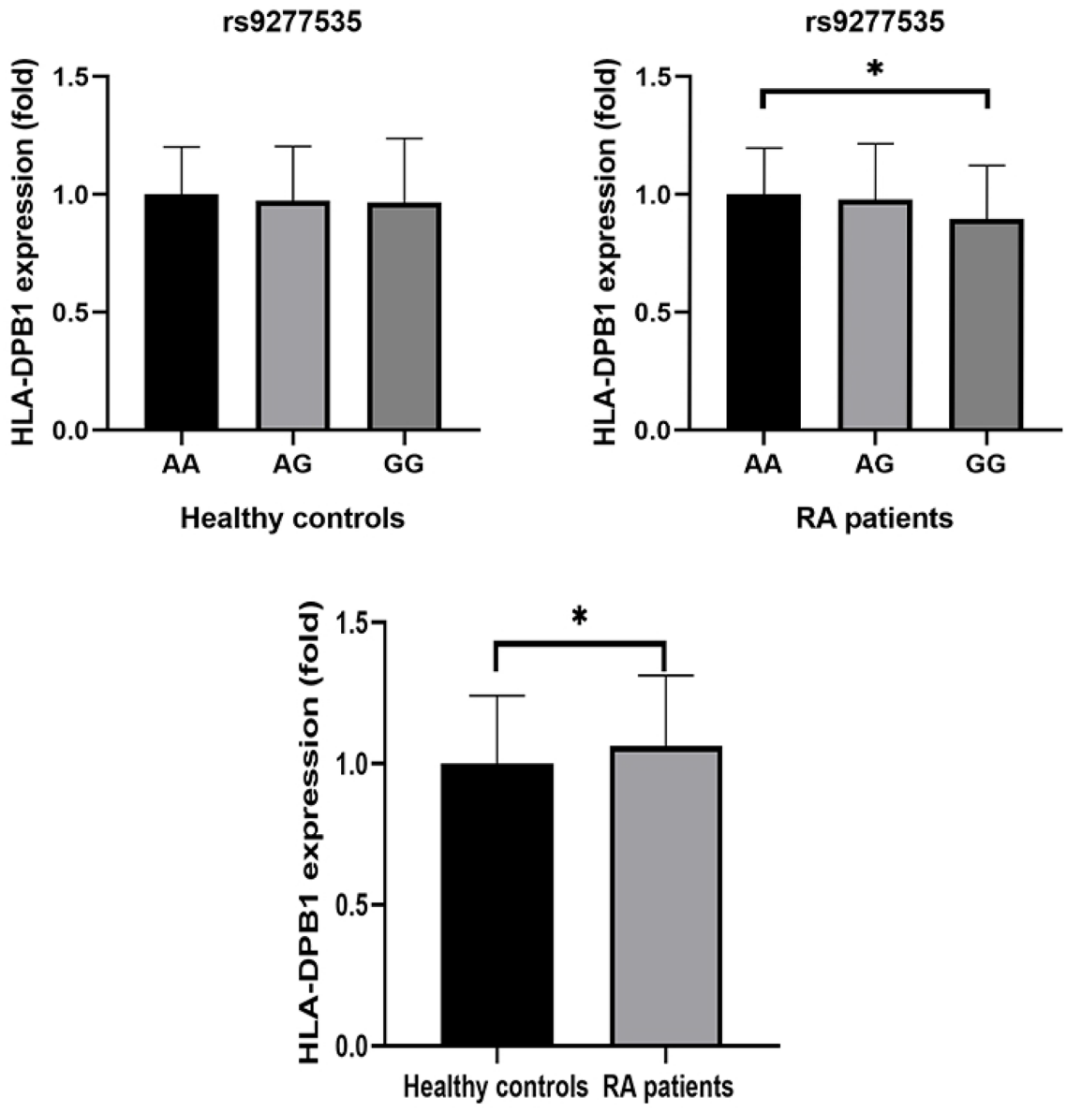
Comparison of relative HLA-DPB1 mRNA expression between RA patients and healthy controls with different genotypes.

Average HLA-DPB1 plasma levels were higher in RA patients than in healthy controls (Table 1). We then examined differences in plasma HLA-DPB1 levels depending on HLA-DPB1 rs9277535 genotype and found that HLA-DPB1 levels were significantly lower in those with the GG genotype than in those with the AA genotype (Figure 1). This relationship between HLA-DPB1 rs9277535 genotype and HLA-DPB1 plasma levels was similar in both RA patients and healthy controls (Table 6). Finally, RT-qPCR analysis indicated that HLA-DPB1 expression was lower in those with the GG genotype than in those with the AA genotype (*P* < 0.05) (Figure 2).

**Table 6:** The associations between *HLA-DPB1 rs9277535* polymorphism and plasma levels of TLR4.

	**TLR4 plasma levels (pg/ml)**		***P* value**
	**RA patients (n=170)**	**Control subjects (n=170)**	
AA	166.28 ± 41.89	142.60 ± 50.48	**0.021**
AG	165.80 ± 51.86	146.27 ± 50.46	**0.016**
GG	149.78 ± 38.35	146.40 ± 40.94	0.689
AA + AG + GG	161.52 ± 45.73	145.59 ± 48.04	**0.002**

## DISCUSSION

In this study, we examined the relationship between HLA-DPB1 rs9277535 polymorphism and RA risk in Chinese individuals. The results indicated that HLA-DPB1 rs9277535 polymorphism down-regulated the risk of RA, especially in women and in those at least 55 years old. In addition, the AG and GG+AG genotypes were associated with CRP status, ACPA status, and ESR in RA patients when the AA genotype was used as the reference group. Moreover, average HLA-DPB1 plasma levels were higher in RA patients than in healthy controls, although HLA-DPB1 plasma levels and mRNA expression were lower in GG genotype carriers than in AA genotype carriers.

Rheumatoid arthritis (RA) is a complex genetic disease associated with many major histocompatibility complex (MHC) genetic factors [14]. The MHC region is divided into Class I (HLA-A, HLA-B, and HLA-C), Class II (HLA-DRB1, HLA-DQB1, and HLA-DPB1), and Class III (C2 et al.) groups [15]. The Class II HLA-DPB1 gene locus, which encodes proteins that bind to small antigen peptides and carry them into the cell surface for presentation to CD4+ T cells, has crucial effects on the immune system [16]. This HLA genomic region is therefore an important regulator of susceptibility to autoimmune diseases such as RA. Yarwood et al. demonstrated that certain HLA-DPB1 variants can be used to identify individuals at risk of developing RA [14]. Moreover, Furukawa et al*.* identified an association between DPB102:01 and ACPA (+) RA in Japanese populations [17]. In addition, O'Brien et al. reported that the A allele at rs9277535 was associated with decreased HLA-DPB1 mRNA levels in the normal human liver [18].

Several recent studies have examined associations between HLA-DPB1 rs9277535 polymorphism and risk of autoimmune diseases. Yang et al. demonstrated that this locus was associated with the risk of IgA nephropathy in a Southwest Chinese population [19]. According to Song et al., the rs9277535 AA genotype was associated with decreased risk of Hepatitis B virus infection in a Yunnan population [20]. Moreover, rs9277535 might be a valuable predictive factor for HBeAg-negative chronic hepatitis B cases [21]. In addition, Zhang et al. found that this polymorphism was associated with Systemic lupus erythematosus (SLE) and with levels of some inflammatory cytokines in SLE patients [22]. Liu et al. also examined associations between HLA-DP polymorphisms and ankylosing spondylitis (AS) in Southwest China and reported that rs927735 was unrelated to risk of AS [23]. The HLA-DPB1 rs9277535 polymorphism may play a similar role in RA angiogenesis as that observed for other autoimmune diseases.

A previous study of 254 Chinese RA patients and 391 control subjects demonstrated that the HLA-DPB1 rs9277535 locus is associated with an increased risk of RA and with elevated serum anti-CCP levels [15]. However, we found here that the HLA-DPB1 rs9277535 polymorphism was associated with decreased susceptibility to RA in Chinese individuals. The results of other studies might help explain this inconsistency. First, Huang et al. reported a frequency of 42.9% for the A allele at rs9277535; this frequency was significantly higher in our study. According to data from the 1000 Genomes database, the A allele frequency at this locus is 46.1% in the Beijing Chinese population. Here, we observed an A allele frequency of 48.3%, which is close to the frequency reported in 1000 Genomes and indicates that our patient population was representative of the larger Chinese population. Second, dietary and other regional differences may contribute to differences in the association between this polymorphism and RA risk in different studies. Third, the relatively small sample sizes included in existing studies may reduce the accuracy of these risk assessments.

Our results indicate that HLA-DPB1 is a useful gene for determining RA risk in the Chinese population. Specifically, HLA-DPB1 rs9277535 polymorphisms were associated with reduced risk of RA in women, in CRP-positive and RF-positive individuals, and in those with DAS28 ≥ 3.20 or ESR ≥ 25, demonstrating that Chinese individuals exhibiting these characteristics are less prone to RA. Next, we assessed the association between rs9277535 polymorphism and HLA-DPB1 plasma levels and mRNA expression. The data indicated that HLA-DPB1 plasma levels were significantly lower in RA patients than in healthy controls. Furthermore, HLA-DPB1 plasma levels and mRNA expression were lower in those with the GG genotype than in those with the AA genotype. Taken together, these results suggest that HLA-DPB1 gene polymorphism may affect HLA-DPB1 expression and serum levels, resulting in a decreased risk of RA.

Some limitations of this case-control study should be considered when interpreting the results. First, selection bias is inevitable for a case-control study based on a limited number of hospitals. Second, gene-environment and gene-gene interactions were not explored. Third, the molecular mechanisms underlying associations between HLA-DPB1 rs9277535 polymorphism and RA risk should be examined in subsequent studies.

## CONCLUSIONS

In brief, the HLA-DPB1 rs9277535 polymorphism down-regulated the risk of RA in the Chinese Han. Thus, in-depth studies on more individuals in other populations should be conducted.

## MATERIALS AND METHODS

### Subjects

The study protocol was approved by the Ethics Committee of Nanjing Medical University (Nanjing, China). Written informed consent was obtained from all included subjects. The Ethics Committee of Nanjing Medical University number was “[2013] scientific research 002-01”. RA patients were consecutively recruited by the Affiliated Changzhou No.2 People’s Hospital of Nanjing Medical University (Changzhou, China) and the Changzhou First Hospital (Changzhou, China) from September 2010 to January 2019. All RA cases satisfied the 2010 ACR/EULAR classifying criteria for RA. Healthy controls were randomly selected from the same institutions during the same time. Trained personnel interviewed all subjects using a pre-established questionnaire to acquire demographic information and RA risk factor data. When the interview process was complete, 2 mL of peripheral blood was collected from each subject in vacutainers and then transferred to test tubes with ethylenediamine tetra-acetic acid (EDTA).

Genomic DNA was isolated from whole blood using the QIAamp DNA Blood Mini Kit (Qiagen, Hilden, Germany). Genotyping was performed by matrix-assisted laser desorption/ionization time-of-flight mass spectrometry (MALDI-TOF MS) (Kindstar Global, Wuhan, China) using the MassARRAY system according to a previously described protocol.

Blood plasma concentrations of HLA-DPB1 were measured in 170 RA patients and 170 randomly selected healthy controls using an enzyme-linked immunosorbent assay Kit. HLA-DPB1 concentrations were determined based on a standard curve and analysis was conducted according to the manufacturer’s guidelines. RNA was extracted from the blood samples using the RNeasy Mini-kit (Qiagen, Valencia, CA, USA) in accordance with the manufacturer’s guidelines. Reverse transcription was then performed using the iScript cDNA synthesizing tool (Bio-Rad, Hercules, CA, USA) according to the manufacturer’s protocol. Complementary DNA samples were aliquoted and stored at -80° C. HLA-DPB1 gene expression was assessed using SsoAdvanced™ Universal® SYBR Green Supermix (Bio-Rad, Hercules, CA, USA) and an MX4000 Stratagene for detection in accordance with the manufacturer’s guidelines. The PCR amplification protocol was as follows: an initial hold at 95° C for 3 min followed by 35 cycles of 95° C for 30 s, 60° C for 30 s, and 72° C for 30 s. Relative expression was quantified using the 2-ΔΔCT method.

### Statistical analyses

Associations between HLA-DPB1 rs9277535 polymorphism genotypes and demographic and other variables were assessed in chi-squared tests. Relationships between HLA-DPB1 rs9277535 genotypes and RA risk were assessed by calculating odds ratios (ORs) and 95% confidence intervals (CIs) via logistic regression analysis and using crude ORs. Based on a goodness-of-fit chi-squared test, Hardy–Weinberg equilibrium (HWE) was determined to compare observed and expected genotype frequencies. Non-parametric tests were used to assess differences in HLA-DPB1 blood plasma concentrations based on HLA-DPB1 gene polymorphism. Student’s t-tests and ANOVAs were performed using GraphPad Prism 5.0 (GraphPad Software, LaJolla, CA, USA). Other statistical analyses were performed using SAS software (version 9.1.3; SAS Institute, Cary, NC, USA).

### Data availability statement

The data sets used in the current study are available from the corresponding author upon reasonable request.
